# Mating dynamics of a sperm-limited drosophilid, *Zaprionus indianus*

**DOI:** 10.1371/journal.pone.0300426

**Published:** 2024-03-25

**Authors:** Jennifer M. Gleason, Barnabas Danborno, Marena Nigro, Henry Escobar, Micalea J. Cobbs

**Affiliations:** 1 Department of Ecology and Evolutionary Biology, University of Kansas, Lawrence, Kansas, United States of America; 2 Department of Anatomy, Faculty of Basic Medical Sciences, Ahmadu Bello University, Zaria, Nigeria; 3 Undergraduate Biology, University of Kansas, Lawrence, Kansas, United States of America; University of Massachusetts, UNITED STATES

## Abstract

When males have large sperm, they may become sperm limited and mating dynamics may be affected. One such species is *Zaprionus indianus*, a drosophilid that is an introduced pest species in the Americas. We examined aspects of mating behavior in *Z*. *indianus* to determine the senses necessary for mating and measure female and male remating habits. We found that vision is necessary for successful copulation, but wings, which produce courtship song, are not needed. Males need their foretarsi to successfully copulate and although the foretarsi may be needed for chemoreception, their role in hanging on to the female during copulation may be more important for successful mating. Females that mate once run out of sperm in approximately five days, although mating a second time greatly increases offspring production. Females do not seem to exert pre-mating choice among males with respect to mating with a familiar versus a novel male. Males are not capable of mating continuously and fail to produce offspring in many copulations. Overall, females of this species benefit from polyandry, providing an opportunity to study sexual selection in females. In addition, the dynamics of male competition for fertilizing eggs needs to be studied.

## Introduction

Large sperm is an investment by males in offspring production. Selection for large sperm in drosophilids is associated with sperm competition because large sperm may displace small sperm in the female reproductive tract [1, 2]. Because large sperm are an energetic investment, males may become sperm limited if they are not able to continuously produce sperm, resulting in a change in the operational sex ratio thus the opportunity for sexual selection, particularly on the female, may increase with sperm size.

In this study, we examined the mating dynamics of a drosophilid with large sperm, *Zaprionus indianus*. Although it has a different genus name, phylogenetically the genus *Zaprionus* is embedded within the genus *Drosophila*, more closely related to *Liodrosophila* and several other genera, as well as the clade containing the immigrans, quinarian, cardini, tripuntata and funebris groups, than to other major subgenus *Drosophila* groups such as the repleta and virilis groups [3]. A native of Africa, the species invaded North and South America starting in 1999 [4–6]. Our study used a strain collected in Kansas. The species was first collected in Kansas in 2012, though in some years we have failed to collect it [7], implying that *Z*. *indianus* reinvades the region when it cannot overwinter [reviewed in 8]. The species is a pest of figs but feeds on more than 80 host plant species [reviewed in 8] with a resource breadth wider than the cosmopolitan species *Drosophila melanogaster* [9].

The species has large sperm, longer than the body length at approximately 5 mm compared to that of *Drosophila melanogaster* at about 1.8 mm [10]. The sperm of *Z*. *indianus* are not giant sperm; the record in *Drosophila* is that of *D*. *bifurca* at ~58 mm [11]. Male *Z*. *indianus* do not produce many progeny: offspring production per day is about 25 and is highly temperature dependent [10].

*Zaprionus indianus* has less sexual dimorphism than *D*. *melanogaster*, though females are bigger than males [12]. Both males and females have the longitudinal zebra-like stripes that characterize the genus [13, 14]. Overall populations have an even sex ratio though females develop faster than males so that the first 72 hours of progeny eclosion is female biased [15].

Female *Z*. *indianus* have an unusual behavior. When approached by males, unreceptive females perform a shaking behavior, which is termed “rocking” in other species [16]. After copulation, females start rocking and performing other refusal signals for at least six hours post copulation [16]; the behavior may disappear within a day (pers. obs). Courtship in this species is brief, lasting seconds to a few minutes [17]. In courtship, a male approaches a female, grapples with her, and produces an acoustic signal (song) through wing vibration [16]. Male courtship songs are brief but necessary because a wingless male is not accepted by females [17]. A female may duet, responding with her own song if receptive, or produce a whining sound while rocking if unreceptive [17]. If not rejected, the male grasps the female’s abdomen with his fore- and mid-tarsi and pushes her wings aside with his head to mount. While mounting the male produces another song with different characteristics from the courtship song [17]. Copulation usually, but does not always, follow mounting [16]. Copulation lasts 2–4 minutes [16].

We explored aspects of courtship and mating behavior in this species to understand the sensory modalities that contribute to successful mating, the choices that females make, if females become sperm limited after mating, and how repeated mating affects male courtship behavior. All together the behaviors observed imply that sperm size affects the dynamics of mating.

## Materials and methods

### Strain and culturing

The strain of *Zaprionus indianus* used was established in 2012 from 16 individuals collected in Topeka, Kansas by Suegene Noh. The strain was cultured on standard cornmeal-molasses food in 6 oz bottles with about 100 individuals per bottle at 25°C on a 12:12 hr Light:Dark cycle. Subcultures for collecting unmated flies for experiments were established with about 20 adults in 25 mm x 95 mm vials (hereafter, large vials) with food. Unmated flies were collected within 8 hours of eclosion and housed in single sex groups of up to 10 in 16 mm x 95 mm vials (hereafter, small vials) with food. Experiments were conducted with at least 6-day old adults because females reach sexual maturity at 5 days [12].

### Counting larvae

In experiments in which the progeny production was measured, females were allowed to lay eggs on food in a large vial for an experiment specific amount of time. Larvae were floated out of the food using 20% sucrose in water before counting.

### Dependence on light for mating

To determine if the species can mate in the dark, a 10-day old unmated female was paired with a single unmated male of the same age in a small vial. The pairs were kept either on a standard 12:12 hour light:dark cycle or in the dark continuously for nine days at 25°C. Pairs placed in the dark were set up under red light so that the pair was never exposed to light. At the end of the incubation, the vials were examined for the presence of larvae to determine if the pair mated.

### Dependence on wings for mating

To determine the effect of wings on mating, unmated flies were collected as above and one day before the trials, the flies were subjected to CO_2_ anesthesia. Wings were removed from half the males and half the females by cutting the wings close to the body. The following day males and females were paired in a mating chamber and video recorded. All possible pairs were recorded on the same video (winged females and males (control), wingless females with winged males, winged females with winged males, and both wingless). Videos were recorded for 15 minutes or until the last copulation was completed, whichever occurred last. Videos were assigned a random number and subsequently scored blindly. Courtship latency was scored as the time from when the last individual was added to the chamber to when the male started courting. Courtship duration was the time from the initiation of courtship until the start of copulation. Copulation duration was the time from the start of copulation until the end of copulation.

### Role of foretarsi in mating

To determine the effect of foretarsi on mating, unmated males and females were collected as above. One day before the experiment, males and females were each assigned to one of two treatments: removal of foretarsi and controls. All individuals were anesthetized with light CO_2_ for the same amount of time. The first five tarsal segments were removed with micro dissection scissors. All individuals were 7–11 days old.

The following day, within two hours of lights on, all four combinations were placed in separate small vials: a control male with a control female, a tarsi-less female with a control male, a control female with a tarsi-less male, and both sexes lacking tarsi. Pairs were observed up to one hour and the time when courtship started, copulation started, and copulation ended were recorded. From these, courtship latency, courtship duration, and copulation duration were calculated as above.

### Sperm limitation

Pairs of unmated males and females in a small vial were observed copulating and the duration of copulation was recorded. Males were then discarded and the females were moved to a fresh vial daily for five days. One week after a vial was established, the number of larvae and pupae was counted as above.

### Progeny production with one or two matings

To determine the retention time of sperm and the effect of a second mating on female fecundity, females were collected as unmated flies as above before being divided into two treatments:

*Single matings*: A 6–11 day old female was place with a similarly aged male in a large vial and observed until copulation occurred or one hour passed. Males and females that did not mate were discarded. After copulation the male was discarded and the female remained in the vial. Every 24 hours the female was moved to a new vial for ten days. The number of larvae present were counted after one week for each vial by floating the larvae out of the food as above.

*Two matings*: For females that were mated a second time, the initial two days proceeded as with the single mating females except the male was retained. After 48 hours, the male was reintroduced to the female. After copulation was observed, or one hour had passed, the male was removed and the female was moved onto new food every 24 hours for eight more days before counting all the progeny one week after a vial was established.

### Female choice

In the first female choice experiment, an unmated female was given the choice for mating between a male with whom she had mated previously and a novel male. Unmated males and females were collected within 6 hours of eclosion and housed in small vials in groups of up to 10. When 7–9 days old, males were randomly assigned to two conditions: familiar and novel. A female was placed in a small vial with a single male from the familiar group. The two were observed for an hour and if mating did not occur, they were discarded. If mating occurred, the male was removed. Half of the familiar males were placed on food containing blue food dye and half of the novel males were placed on food without food dye. Approximately 24 hours later, the familiar male was returned to the vial with the female and a novel, unmated male of the opposite dye state was also added. The male that copulated first within an hour was recorded.

Because the first experiment overwhelmingly favored familiar males, we performed another experiment. Unmated females, 7–10 days old were mated as above and her male was retained. Half of the females were discarded. On the second day, each female that was kept was presented with both the male with whom she mated the day before and one who mated with another female the day before. The male that copulated first within an hour was recorded.

### Male remating rate

To determine the effect of mating on subsequent copulations, males were provided with a series of females and the number of times he mated and his progeny production with each female was measured. Unmated males and females were collected as above. A day prior to trials, females were assigned to one of four marking groups: right wing cut, left wing cut, both wings cut, or no wings cut. Cuts were made with fine scissor and removed just the distal portion of the wing. Markings were necessary to track the females and their progeny.

When a male was 7 days old, he was placed with four females of the same age with different wing markings for 15 minutes in a 16 mm diameter mating chamber. The mating chamber was video recorded. After 15 minutes the male was removed and placed with four new females. This was repeated two more times for a total of four sets of four females in a single day (16 females total). On each of the next three days, the series of sets was repeated, each time with new females (64 females total over all days). All females were placed in large vials to lay eggs for one week before they were discarded. The number of larvae was counted at one week using sucrose floats as above.

Each video was assigned a random number for subsequent blind scoring. Videos were scored for the identity of each female that was mounted and the duration of the mounting. The number of larvae for each female was merged with the duration of copulation for subsequent analysis.

## Results

### Dependence on light for mating

When kept in continuous darkness, the proportion of male-female pairs producing larvae was only 0.04 (N = 100) whereas the proportion producing larvae under 12:12 hour light:dark conditions was 0.65 (N = 100). Darkness greatly suppressed mating (two tailed Fisher’s Exact Test, *P* = 1.27 x 10^−22^). Vials kept in the dark had eggs indicating that the suppression was on courtship success and not on laying ability.

### Dependence on wings for mating

To determine if wings, predominantly through the production of courtship song, affected male and female mating success, we compared pairs of wingless females, wingless males, and both wingless with control pairs. Courtship initiation did not differ in any of the treatments from the control treatment with a proportion of 0.68 (both wingless) to 0.89 (female wingless) of males initiating courtship (N = 18 to 20 for each treatment, Fisher’s exact test comparison to control treatment, all *P* > 0.05). Of those males that courted, the proportion of males copulating ranged from 0.81 (control) to 1 (female wingless) thus the treatments did not differ in the proportion copulating (N = 13 to 17 for each treatment, Fisher’s exact test comparison to control treatment, all *P* > 0.05). Courtship latency, courtship duration and copulation duration did not differ among the treatments (ANOVA, F_3,55_ = 0.50, P = 0.68; F_3,47_ = 1.50, P = 0.23; F_3,47_ = 0.53, P = 0.66, respectively, S1 Fig).

### Dependence on foretarsi for mating

**Fig 1:**
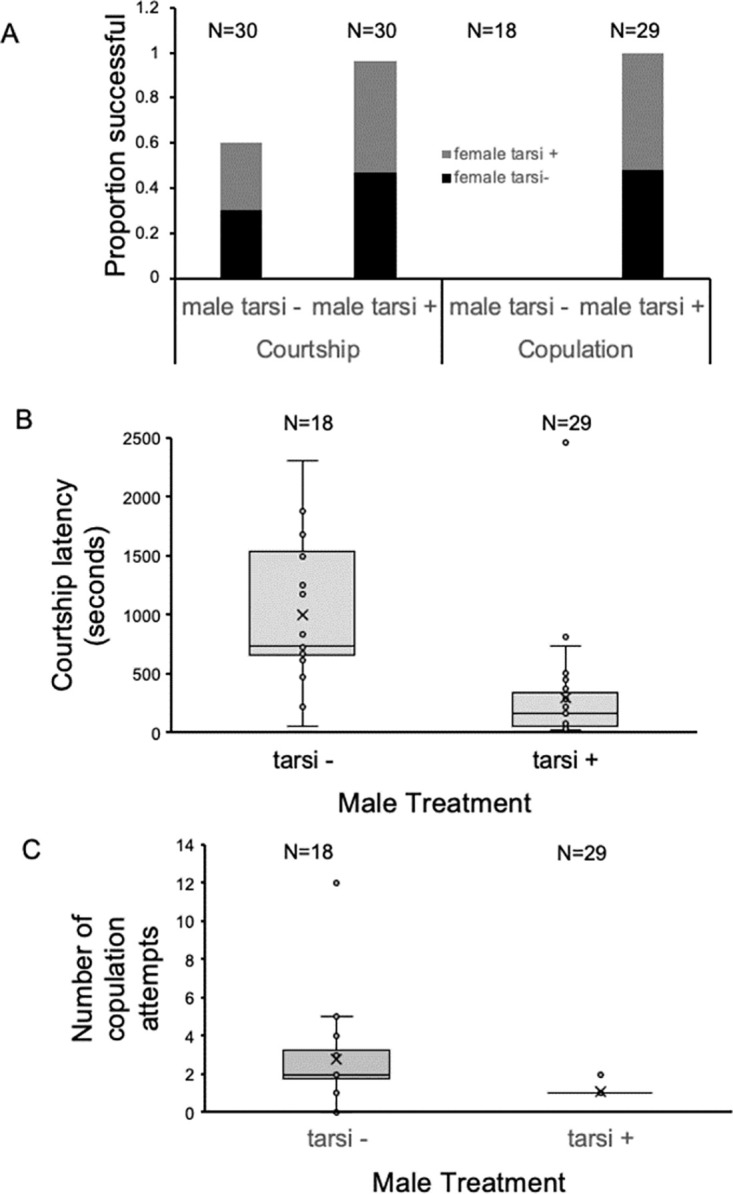
The effect of foretarsi removal on mating parameters. A. Removal of tarsi from males decreases male mating success. Males that lacked foretarsi (tarsi-) were less successful at copulation than males with foretarsi (tarsi+). When males lacked foretarsi, they initiated courtship (left side) less often than when males had foretarsi, independent of the presence (gray, N = 15)) or absence (black, N = 15) of foretarsi on females (N = 30 for male state, Fisher’s Exact Test compared to males with foretarsi, independent of the foretarsi on females, *P* = 0.0001). Of those that courted, males without foretarsi failed to copulate whereas all males with foretarsi that initiated courtship copulated. B and C. All data points are graphed. Boxes represent the middle two quartiles, X designates the mean, the horizontal bar is the median. B. The presence of tarsi positively influenced courtship. Among the males that initiated courtship, males without tarsi took longer to initiate courtship than males with tarsi (T-test, *P* = 6.95 x 10^−5^). C. Among the males that courted courtship, males without foretarsi (tarsi-) still attempted copulation, though they were unsuccessful. Thus, the number of copulation attempts for males without foretarsi was greater than that of males with foretarsi (tarsi+; T-test, *P* = 0.001). Males with foretarsi only attempted copulation 1 or 2 times, including when they successfully copulated.

To determine how foretarsi affect mating success, we compared pairs of normal males withforetarsi-less females, normal females with foretarsi-less males, and both sexes lacking foretarsi with control pairs. Removal of foretarsi from females did not change any parameters of mating from that of females with foretarsi, so for all analyses results were grouped by whether or not the males had foretarsi. When males lacked foretarsi, they initiated courtship less often than when they had foretarsi (Fig 1, Fisher’s exact test, *P* = 0.0001). Males without foretarsi failed to copulate whereas nearly every male with foretarsi was successful (29 out of 30, Fig 1A). Males without foretarsi were substantially delayed in initiating courtship (Fig 1B, T-test, *P* = 6.9 x 10^−5^) and attempted copulation more than males with foretarsi (Fig 1C, T-test, *P* = 0.001), mostly because all males with foretarsi who attempted copulation were successful on their first attempt (one male required two attempts, Fig 1C). Thus lack of foretarsi substantially hampered a male’s ability to hold onto the female for copulation.

### Sperm limitation and copulation duration

**Fig 2:**
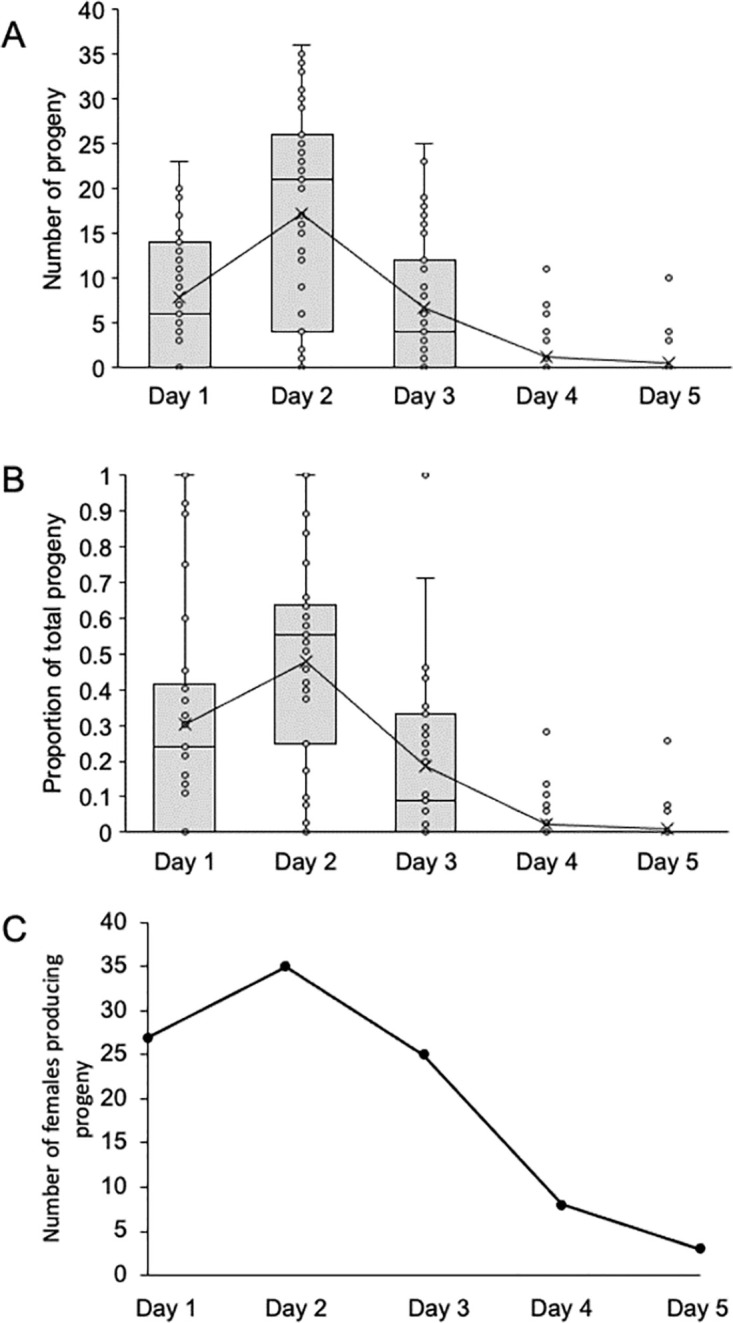
Females mated once have peak production on the day after mating. Females were mated with a single male on Day 1 and the number of progeny produced for each of five days were counted. Only females that produced any progeny across any of the five days are included (N = 39). A. Number of progeny produced each day. Females varied in progeny production, with the most progeny produced on the second day. Progeny production approached zero by day 5. Boxes represent the middle two quartiles, X designates the mean, the horizontal bar is the median. The line connecting the boxes follows the mean. B. Proportion of total progeny. Some females only produced progeny on a single day, but the mean proportion of total progeny produced peaks on the second day. Most females were not producing progeny by day 5. Boxes as in A. C. Number of females producing progeny each day. Nearly all (35 out of 39 total) females produced progeny on the second day and few produced any on day 5.

To determine how long females produce fertile eggs after mating, pairs of males and females were observed mating and then the progeny produced by each female was determined for each day after mating. Of the 46 pairs observed to copulate, seven did not produce any progeny over the five days that the females laid eggs. Copulation duration was short (109.96 ± 7.47 (s.e.) seconds) and was not related to progeny production (S2 Fig, R^2^ = 0.003, *P* = 0.72). Progeny production peaked on the second day for the number of progeny produced (Fig 2A), the proportion of progeny produced over all days (Fig 2B) and the number of females producing progeny (35 of 39 females, Fig 2C). Most females did not produce any progeny on the fifth day (36 of 39 females, Fig 2C). Total progeny production was low (27.98 ± 2.81 s.e.) even without individuals that did not produce any progeny (range 3–61 progeny, mean 33.00 ± 2.60 s.e.). Of those females producing at least one offspring, no female laid eggs all five days (range 1–4 days; mean 2.51 ±0.14). Progeny production was continuous; no female failed to produce progeny on a day between two successful days.

### Progeny production with two matings

**Fig 3:**
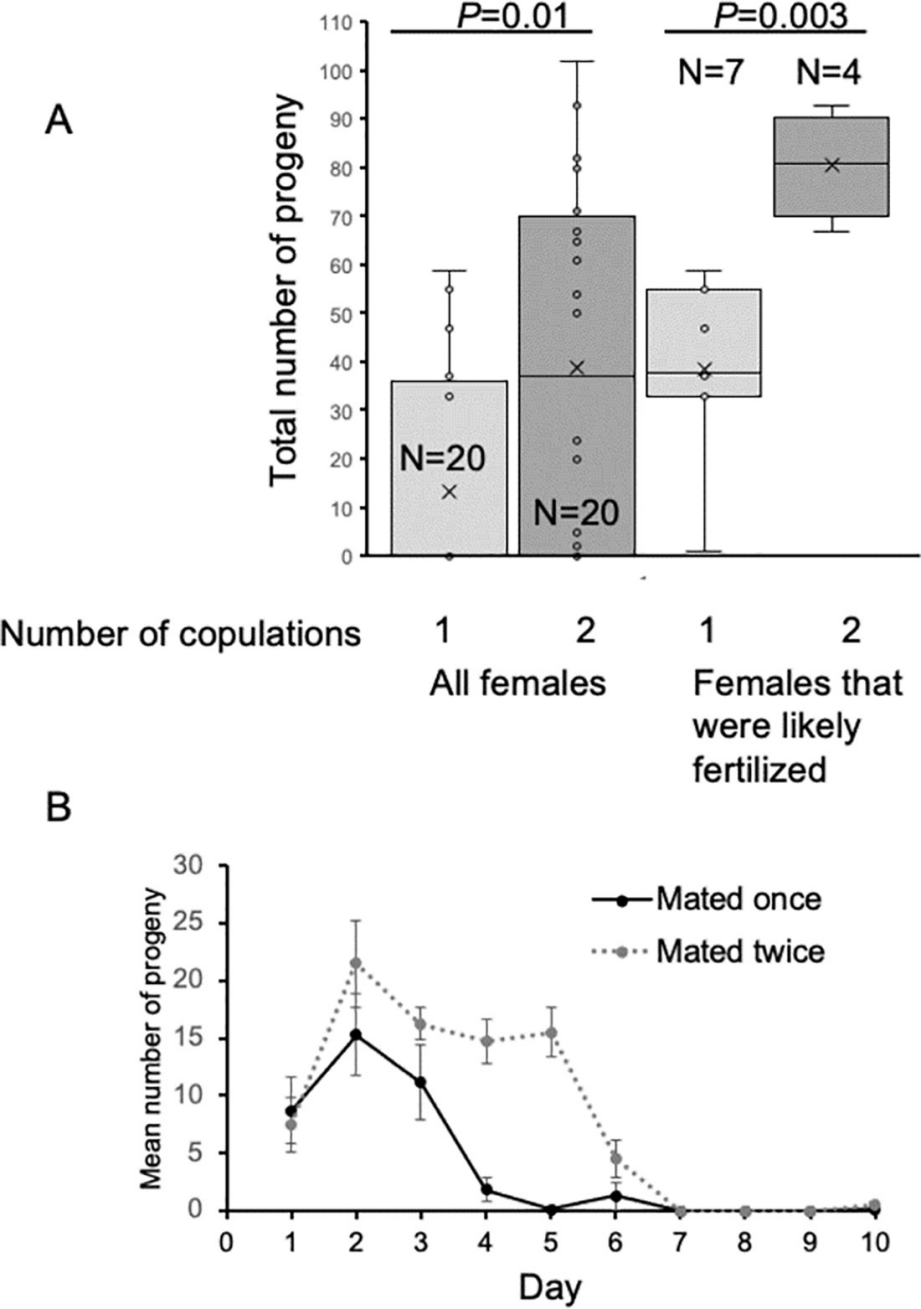
Progeny produced by females that mated once or twice. A. Total progeny produced in one or two copulations. Females were exposed to one or two males. In the plot on the left (All females) the total number of progeny produced is graphed. Many of the matings did not result in fertilization, but the total progeny produced was still significantly different if the female was observed to mate with one male or two (T-test, *P* = 0.01). The number of successful fertilizations was inferred (see text) and is plotted on the right side. Again, females exposed to two males produced significantly more progeny than females exposed to a single male (T-test, *P* = 0.003). B. Progeny production by day. The progeny produced each day following exposure to one or two males. Only females inferred to be fertilized from each mating are plotted (see text). The mean number of progeny (± standard error) are plotted for each day. All females were mated with a male on day 0. Females that were mated with a second male were exposed to that male on day 2 (N = 7 for exposure to one male, N = 4 for exposure to two). The days that the number of progeny produced differed between the two treatments are marked with an asterisk (*, T-test, day 4 *P* = 0.0005, day 5 *P* = 0.00007).

To understand the advantages of mating twice, the progeny production of females that mated once were compared to that of females that mated twice. All females were observed to copulate with the males. As with the previous experiment, some females that were observed copulating failed to produce progeny. Only 7 of the 20 females that were exposed to only one male produced progeny whereas 14 of the 20 females exposed to two males produced progeny; the proportions mating with one or two males is the same (Fisher’s Exact Test, *P* = 0.057). Comparing progeny production for all females, exposure to two males resulted in more progeny even with the majority (N = 25 of 40 trials) of females producing no progeny (Fig 3A, T-test *P* = 0.01).

The data were further partitioned by examining the pattern of progeny production. Of the females exposed to one male, the seven females who produced any progeny all had progeny on the first or second day. Presence of progeny in the first two days after mating was then used as a criterion for whether or not the first mating was successful for the females exposed to a second male on the second day. In the group exposed to two males, of the 14 females that produced any progeny, only 8 produced progeny on the first two days, indicating that they were successfully fertilized by the first males. In the set of females (N = 7) exposed to one male that produced progeny, the mean progeny production on day 5 was 0.14 (± 0.13 standard error, Fig 3B). Thus, for the females exposed to two males, progeny production greater than 1 offspring on day 5 was set as the criterion to determine that a second mating was successful. Of the 8 females exposed to a second male that definitely mated with the first male, only four produced progeny on day 5 (mean progeny production: 15.5 ± 2.11 standard error, Fig 2B). Comparing these two sets of females, those who successfully mated twice had more significantly more progeny than those who mated once (Fig 2A, T-test, *P* = 0.003).

Progeny production over time peaked on the second day after the first mating and then dropped off precipitously if the female was not fertilized again (Fig 3B). If the female successfully copulated a second time on day 2, progeny production remained steady for three more days and was significantly higher for females with two fertilizations compared with females with one fertilization on days 4 and 5. For females fertilized by two males, progeny production dropped dramatically on day 6 (five days after the second fertilization) and did not increase.

### Female choice

**Fig 4:**
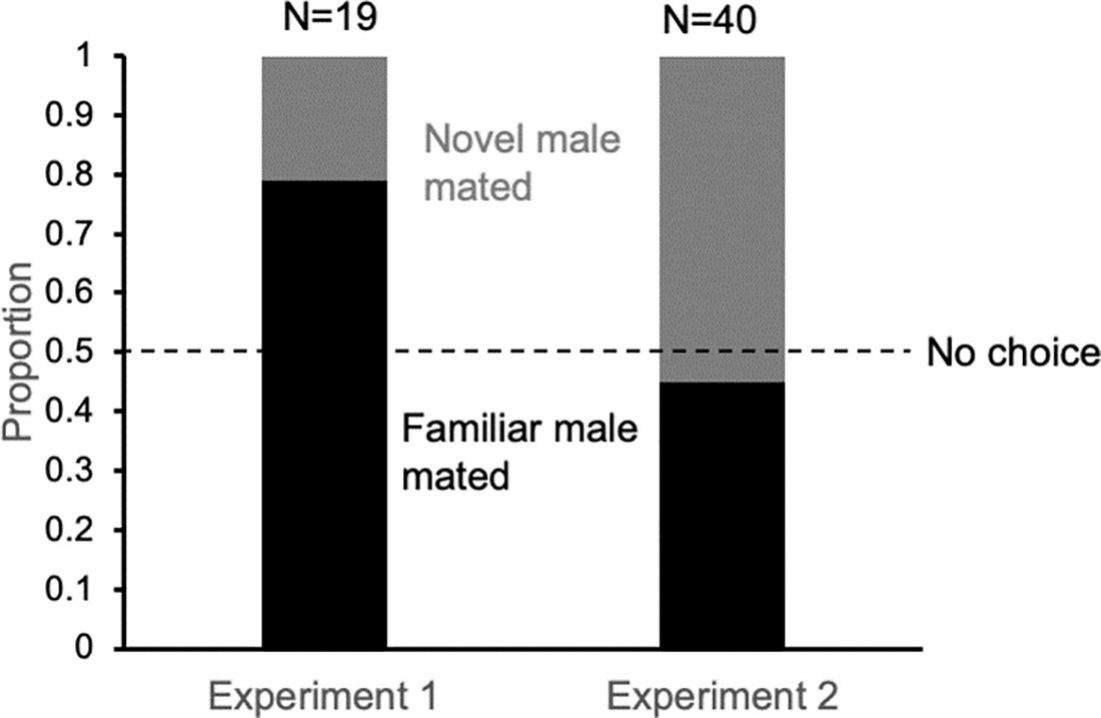
Female choice for second males. Females were given two males one day after mating with a male. The dotted line indicates the expectation if males were equally successful. In Experiment 1, the female had a choice between the male she mated with the previous day (familiar male) and an unmated male (novel male). Familiar males were more successful (Chi-squared test, *P* = 0.012). In Experiment 2, the female had a choice between the male she mated with the previous day (familiar male) and a second male (novel male) that had mated at the same time with a different female. Males were equally successful (Chi-squared test, *P* = 0.052).

Females can increase their progeny production with a second mating, but do they increase diversity in their offspring by preferring a novel male over a familiar male? When females were given a choice between a male with whom she had mated the day before (familiar male) and an unmated male (novel male), the proportion of females mating with the familiar male was 0.79 (N = 19) thus the familiar males were more successful (Chi-squared test, *P* = 0.012; Fig 4, Experiment 1). Because the males did not have the same courtship experience (the novel males were unmated), the experiment was repeated with novel males that had also mated the day before. In this case, the proportion of females (N = 40) mating with the familiar male was 0.44, which was not significantly different from random choice (Chi-squared test, *P* = 0.527; Fig 4, Experiment 2). In each experiment, females did not choose based on whether or not the male was dyed (Experiment 1 Chi-squared test, *P* = 0.819; Experiment 2 Chi-squared test, *P* = 0.20).

### Male remating rate

Twelve males were exposed to 16 females (in sets of four) per day for four days. Males varied in the number of times they were observed to mate (Table 1). Not all copulations produced progeny, thus the data were analyzed both with respect to the total number of observed matings and the number of observed matings that produced progeny. Although many matings failed to produce progeny, per male the number of matings and the number of progeny summed over all four days were highly correlated (S3 Fig, R^2^ = 0.69, *P* = 0.0008 for all matings; R^2^ = 0.67, *P* = 0.001 for only matings producing progeny). Despite this, the number of progeny produced per mating for each male was not related to copulation duration (S3 Fig, R^2^ = 0.0045, *P* = 0.84 for all matings, R^2^ = 0.0018, *P* = 0.90 for only matings producing progeny). Males were highly variable in fecundity if matings with no progeny were excluded (all matings: ANOVA, F_11,131_ = 1.36, *P* = 0.2; matings producing progeny: ANOVA F_11,60_ = 5.11, *P* = 1.4x10^-5^). Similarly, males varied greatly in copulation duration, but only if matings with no progeny were excluded (all matings: ANOVA, F_11,131_ = 1.74, *P* = 0.07; mating producing progeny: ANOVA F_11,60_ = 2.40, *P* = 0.02).

**Table 1:** Summary statistics of male remating experiment (N = 12 males).

	Mean	Standard Error	Range
Number of mating attempts per male	11.91	1.89	5–23
Number of successful mating attempts per male	6.08	1.05	2–13
Number of progeny per male	122.25	27.91	24–375
Number of progeny per mating attempt per male	9.56	1.35	4.60–19.75
Number of progeny per successful mating attempt per male	19.64	2.58	8.20–39.50
Number of progeny per male per day (all 4 days)	30.56	6.98	6–93.75
Day 1 (all sets)	60.08	13.30	0–132
Day 2 (all sets)	21.42	6.48	0–60
Day 3 (all sets)	19.50	5.62	0–50
Day 4 (all sets)	21.25	16.77	0–212
Number of progeny per male per set (all 16 sets)	7.64	1.74	1.50–23.44
Set 1 (all days)	56.25	6.92	8–91
Set 2 (all days)	45.33	15.60	0–154
Set 3 (all days)	13.25	5.41	0–51
Set 4 (all days)	7.42	7.10	0–89
Copulation duration (all observed matings), seconds	106.96	4.76	14–479
Copulation duration (all successful matings), seconds	106.97	4.22	43–256

**Fig 5:**
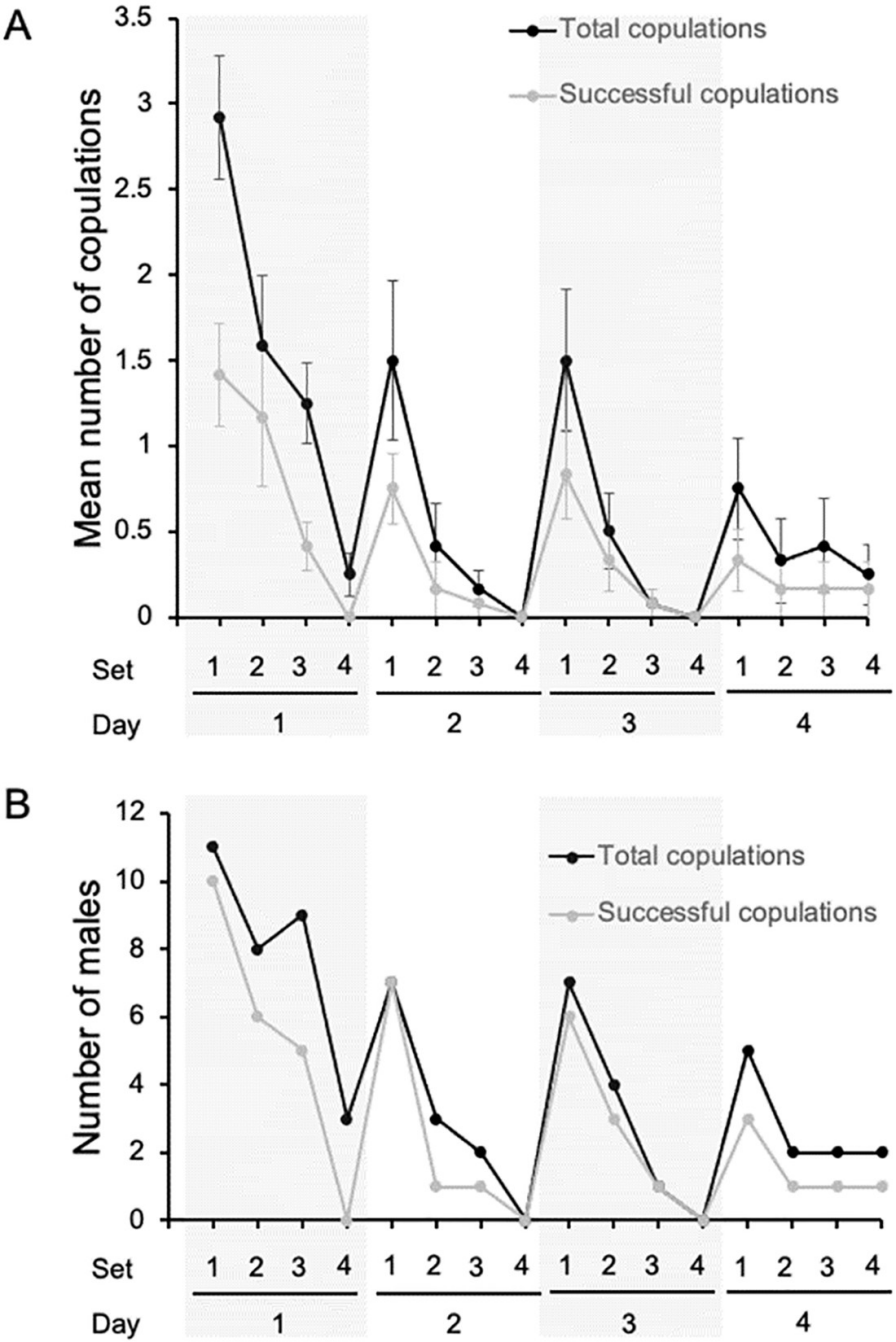
Male mating patterns over all days and sets. Days 1 and 3 are shaded to delimit days clearly. A. Observed matings. Male decreased their attempts across sets each day and across days. Not all matings were successful as measured by progeny production. Observed matings are in black and successful matings are in gray. Values are means with standard errors. B. Number of males observed mating. The number of males observed mating decreased over sets each day and across days. The total number observed is in black and the number that were successful, as measured by progeny production is in gray.

**Fig 6:**
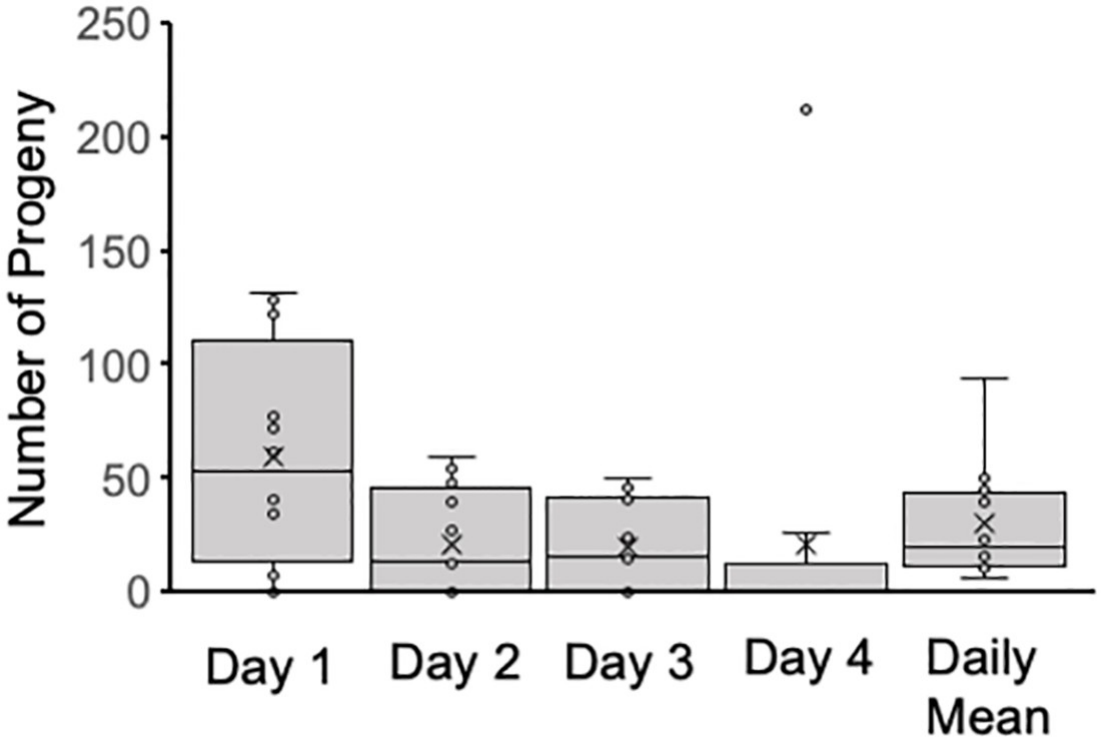
Progeny production per day. The number of progeny that males (N = 12) produced decreased over the time of the male remating experiment, with a daily mean less than the first day and greater than the subsequent days. Boxes represent the middle two quartiles, X designates the mean, the horizontal bar is the median. The number of progeny produced on the first day was higher than that produced on any of the other days (paired t-test, *P* = 0.018, 0.017, 0.048 respectively).

Males attempted and were successful at mating less often across days, and across sets within days with some recovery from the end of one day to the beginning of the next, but not to the level of the first set of the previous day (Fig 5A). At the same time, the number of males attempting mating and being successful decreased such that on the last day, although multiple males attempted to mate, only one was successful in the last three sets (Fig 5B). All males did not mate in any single set, including the first set on the first day. Progeny production tended to decrease over time by day (Table 1, Fig 6). The mean progeny production per day was 30.6 ± 7.3 (s.e.) offspring with more produced the first day (mean 60.1 ± 13.3 offspring) than the other days (Fig 6).

**Fig 7:**
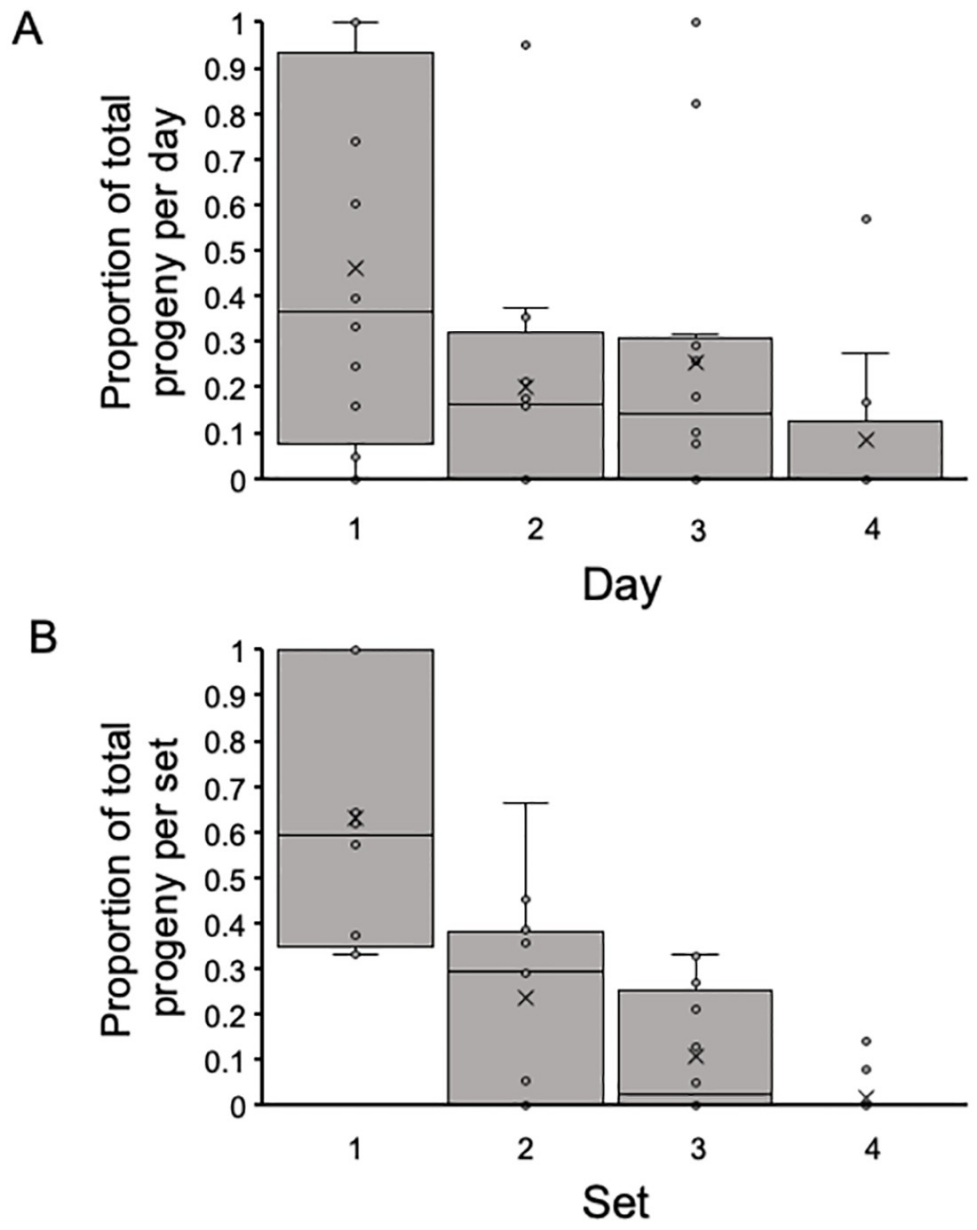
The proportion of total progeny produced by each male by day over all sets (A) or by set over all days (B). The mean proportion of total progeny was highest on the first day and decreased over subsequent days (ANOVA, F_3,44_ = 4.68, *P* = 0.006), though there were males that produced most of their progeny on Days 2 and 3 including one male that produced all his progeny on Day 3. Most progeny were produced in the first sets with sharp declines across sets (ANOVA, F_3,44_ = 17.70, *P* = 1.10 x 10^−7^). Boxes represent the middle two quartiles, X designates the mean, the horizontal bar is the median.

Subsequent analyses of progeny production were performed using the proportion of the total progeny produced by a male in each day and/or set. Day and set affected progeny production (across all sets, Fig 7A, ANOVA, F_3,44_ = 4.68, *P* = 0.006 and all days, Fig 7B, ANOVA, F_3,44_ = 17.70, *P* = 1.10 x 10^−7^). The first day (independent of set) and the first set (independent of day) has the highest proportion of total progeny production.

## Discussion

### Factors contributing to mating success

The substantial decrease in mating success in the dark implies that vision is necessary for successful courtship. This makes *Zaprionus indianus* a type III species, one that requires light to mate, using Grossfield’s classification scheme for light dependency [18]. The species does not have overt visual signals, lacking wing movements such as those found in most spotted wing flies that are necessary for mating success [19]. Nonetheless, the visually striking stripes running from the head through the thorax may provide signals, though these stripes are not sexual dimorphic and exist throughout the genus.

Removing the wings of males did not affect copulation success, implying that courtship song is not necessary. This result differed substantially from previous research [17], which found that wingless males were always rejected by females (though no data was presented). Variation in strains is possible: our strain was from a different geographical location (Kansas) whereas Müller et al. [17] examined three strains from South America. Nonetheless, all strains are from a single invasion of the western hemisphere [20, 21].

We found that males require the ends of their foretarsi for successful copulation. Male courtship was affected by the foretarsi, which is consistent with foretarsi playing a role in chemoreception as in other species that detect cuticular hydrocarbons through their feet and initiate or break off courtship depending on the attractiveness of the individual touched [22, 23]. Males that lacked foretarsi yet still courted tried to copulate repeatedly, but were unsuccessful. Most males with tarsi (all except one) were successful at copulation with their first attempt, which is different from the behavior patterns of other *Drosophila* that may attempt copulation multiple times before being accepted by the female [24]. The foretarsi may be necessary for a male to hold onto a female, who seems to be able to dislodge the male, particularly with rocking behavior. The complete elimination of male mating success with the removal of the terminal foretarsi is different from that found in *D*. *malerkotliana* [25] and *D*. *saltans* [26], both of which mate at a reduced rate when lacking their terminal foretarsi.

For males, another potential factor increasing their reproductive success is experience because previously mated males were more successful at mating than naïve (unmated) males (Fig 4). The foretarsi experiments imply that males need to be able to hold onto the female, thus experience may improve a male’s ability to mount and inseminate a female. An alternative explanation is that the behavior of an experienced male changes with sperm limitation to make a male less aggressive towards a female. Less aggressive, experienced males might be more attractive to females than unmated males. This hypothesis should be tested, but we do not think this is a likely scenario because males were able to successfully inseminate other females within the same day (Fig 5), and many males could inseminate females 24 hours after a first mating. Thus, one mating is not enough to deplete a male fully, though the potential for a change is behavior may exist.

### Sperm limitation, copulation duration, and female productivity

By measuring progeny production over time, we found that females stop producing offspring within five days of mating (Figs 2A and 3B). Because progeny production does not occur again within 10 days (Fig 3C), the likely explanation is not because females experience low ovulation that needs to recover, but rather because they run out of sperm. Remating increases female reproductive potential. Presumably because the males have large sperm, a limited number of sperm are transferred and thus the number of offspring that can result from a single mating is low. In some *Drosophila* species, a female that mates twice does not increase the number of offspring she produces [27, 28]. Failure to increase offspring production implies either sperm competition (the second mate displaces the first male’s sperm or the first mate’s sperm excludes the second’s) or female are selecting sperm and choose the sperm of one male over the other. Two large sperm species, with much larger sperm than *Z*. *indianus*, do not increase offspring production with a second mating (*D*. *bifurca* [28], *D*. *hydei* [29]). In contrast, females that increase offspring production are using the sperm of both males, as implied in *Z*. *indianus*, though this needs to be confirmed by identifying the paternity of the offspring. A further implication is that sperm competition may not exist in this species. Female *Z*. *indianus*, benefit from polyandry and should mate multiply, thus it is surprising that females actively reject suitors after mating. In *D*. *melanogaster* the male transfer proteins in the ejaculate that affect female behavior, lowering receptivity and attractiveness and thereby increasing the male’s reproductive success [reviewed in 29]. Similar effects may be happening in *Z*. *indianus*, decreasing female receptivity after mating.

Peak offspring production occurs two days after copulation followed by a rapid decline if the female does not remate. The peak is two days earlier than seen in the *D*. *simulans* clade (including *D*. *sechellia* and *D*. *mauritiana*), for which the peak is at 4 days followed by a slow decline [30]. *Drosophila simulans* does not become sperm limited, thus can produce offspring continuously. However, in another species that becomes sperm limited, *D*. *bifurca*, females start to lay their eggs five days after mating and do not remate before they lay the full complement [31]. Again, *Z*. *indianus* females lay eggs faster than some other species and need to remate often.

In the experiment in which we measured the offspring production of females mated twice (as well as in the male remating experiment), we observed many copulations in which fertile offspring were not produced. We cannot determine from our experimental design if sperm was transferred and, if transferred, if it went into storage. In *D*. *bifurca*, which has the largest of all sperm, males always transfer sperm but it does not always go into storage [27]. Sperm storage may be under control of the female [32] implying female choice. When females fail to store sperm, they are faster to remate [27]. This is very different than the situation in *D*. *hydei* where females may remate immediately after mating [28].

Copulation duration is brief in *Z*. *indianus* and is not related to offspring production. In other species, notably in *Drosophila melanogaster* subgroup, a minimum time of approximately 4–8 minutes is needed to initiate sperm transfer [33] but likewise, the amount of sperm transferred in not related to copulation duration [34]. Copulation duration in *Zaprionus inidianus* is short implying that sperm transfer is initiated quickly. Even with longer than average copulations, females often did not produce progeny. Given our experimental design, we cannot distinguish among males failing to transfer sperm, a failure to store sperm, and females exerting cryptic choice post mating.

### Female choice

Males have a competitive advantage when they have previous experience with copulation as seen when males that had previously mated were successful more than males that were naïve (Fig 4). Some of the advantage may come from learning how to hold onto the female, as demonstrated by the experiments with and without tarsi. In other species, including *D*. *melanogaster*, males have a competitive mating advantage when they have previous mating experience [35]. Given this scenario, and that male courtship is so brief, females may not be able to exert much choice among males, except through refusal by their rocking behavior, yet unmated females have not been seen to rock (personal observation).

### Male behavior

Through our mate remating experiments, we found that males vary greatly in fecundity, both in the number of eggs fertilized and the ability of the male to court and copulate with females. Male interest in copulation decreased with consecutive matings, but some males copulated with females more often than other males. Male ability to fertilize eggs decreases over time, indicating that males may need time to produce sperm. In addition, not every observed mating resulted in progeny, reflecting what we saw in earlier experiments.

We found a mean male progeny production per day (~31) that is similar to the previously reported 25 per day [10]. The variation in male reproductive capacity from 6 to 63.75 offspring per day (Table 1) is extreme. More work is needed to understand variation in male fecundity.

Our study of *Zaprionus indianus* has opened up many questions about the dynamics of reproductive success in this species. In the future we would like to understand more about why so many matings fail produce progeny. Are males allocating different amounts of sperm to different females? Males are likely becoming sperm limited, but why are some males able to continue producing progeny whereas others stop mating? Given that courtship is minimal in this species, are females exerting choice through postcopulatory means? The answers to these questions may have implications for control of this pest species. Given that females can remate and seem to do so often, methods such as the sterile male technique are unlikely to be effective. Understanding the ecology of *Zaprionus indianus*, particularly the non-fig breeding sites used, may provide insights into reducing the spread of the species.

## Supporting information

S1 Fig The presence or absence of wings does not affect courtship success. All data points are graphed. Boxes represent the middle two quartiles, X designates the mean, and the horizontal bar is the median. A. Courtship latency did not differ, ANOVA F_3,55_ = 0.50, P = 0.68. B. Courtship duration did not differ, ANOVA F_3,47_ = 1.50, P = 0.23. C. Copulation duration did not differ, ANOVA F_3,47_ = 0.53, P = 0.66.
(TIF)

S2 Fig Duration of copulation is not associated with the total number of progeny produced (R^2^ = 0.003, *P* = 0.72). Each mating was timed and progeny were collected for five days post mating. Of the 46 females tested, seven females did not produce any progeny. The minimum time spent in copulation was 28 seconds and the mean was 109.96 ± 27.98 (s.e.) seconds. The mean ± standard error for progeny production was 27.98 ± 2.81 or 5.6 ± 0.56 per day. Including only those days on which progeny were produced, the mean was 12.98 ± 0.79 (N = 39) per day.
(TIF)

S3 Fig Mating events but not duration are correlated with the number of progeny produced. **A**. The total number of matings for each male (N = 12) over four days was positively associated with the total number of progeny produced by each male when all matings were included (blue, P = 0.0008) or when only those producing progeny were included (orange, P = 0.001). B. Progeny production was not related to copulation duration for individual males. Data shown are the mean for each male ± standard error.
(TIF)
