# Supplementary figures and images for: Mating dynamics of a sperm-limited drosophilid, Zaprionus indianus
Source: PLoS One. 2024 Mar 25;19(3):e0300426. doi: 10.1371/journal.pone.0300426 (PMC10962835; doi:10.1371/journal.pone.0300426)

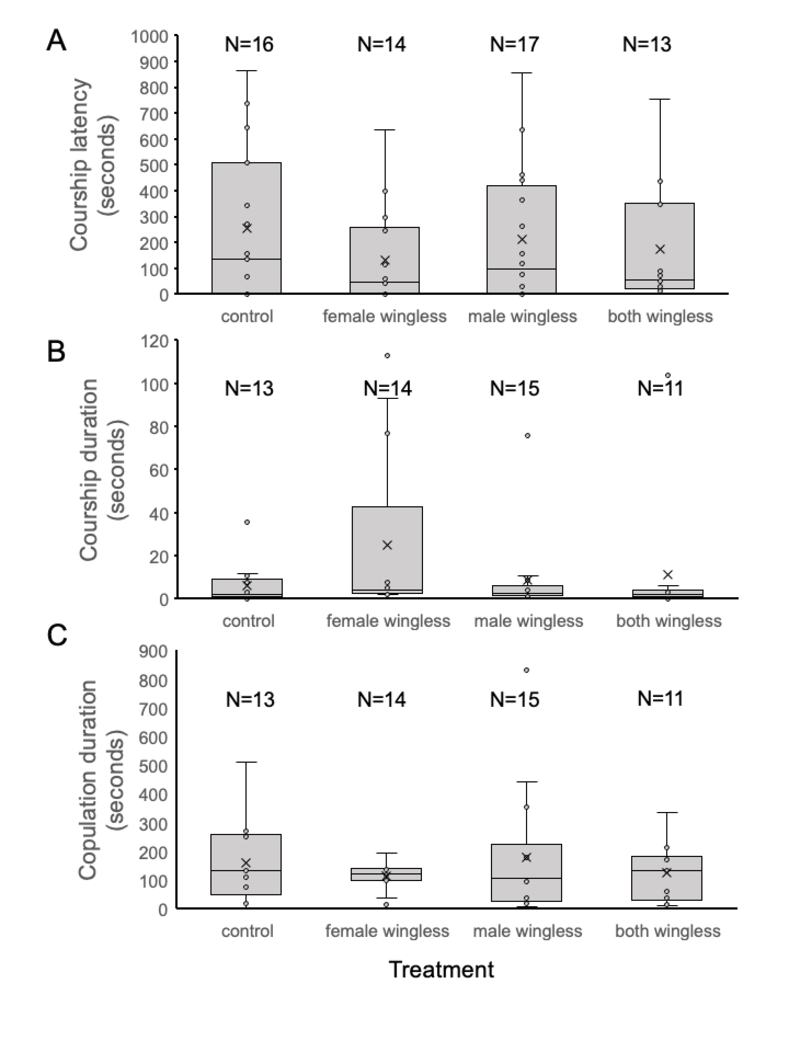

Supplement: S1 Fig — All data points are graphed. Boxes represent the middle two quartiles, X designates the mean, and the horizontal bar is the median. A. Courtship latency did not differ, ANOVA F3,55 = 0.50, P = 0.68. B. Courtship duration did not differ, ANOVA F3,47 = 1.50, P = 0.23. C. Copulation duration did not differ, ANOVA F3,47 = 0.53, P = 0.66. (TIF) [file pone.0300426.s001.tif]

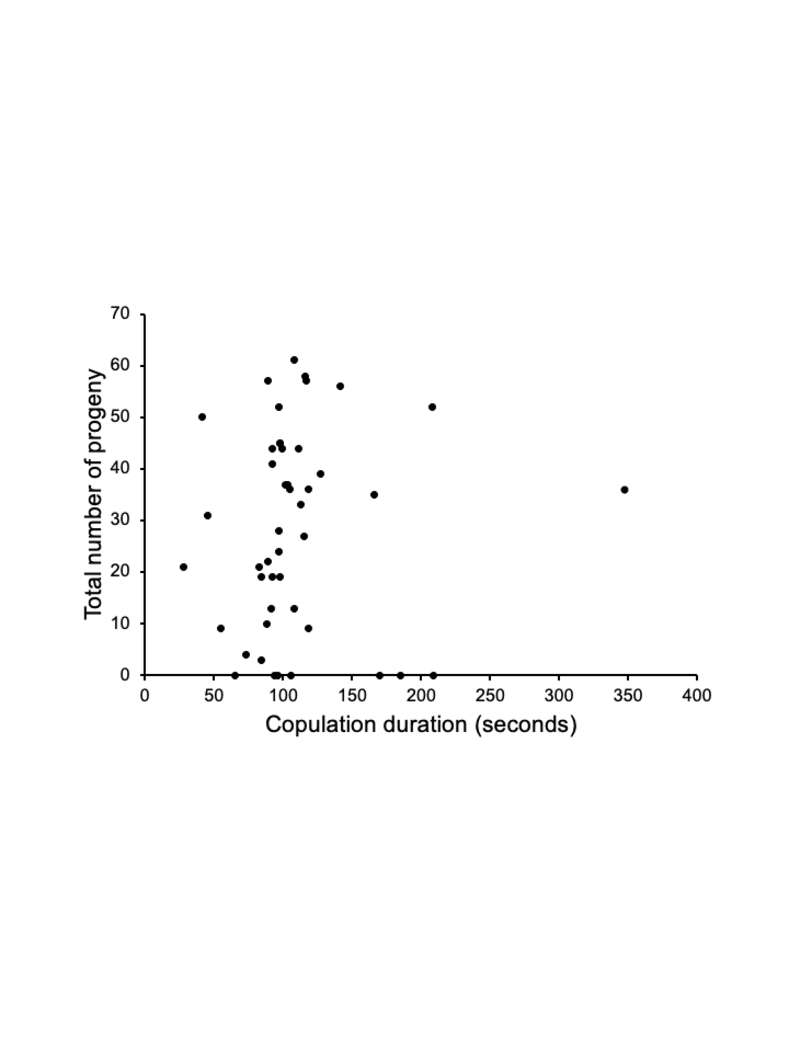

Supplement: S2 Fig — Each mating was timed and progeny were collected for five days post mating. Of the 46 females tested, seven females did not produce any progeny. The minimum time spent in copulation was 28 seconds and the mean was 109.96 ± 27.98 (s.e.) seconds. The mean ± standard error for progeny production was 27.98 ± 2.81 or 5.6 ± 0.56 per day. Including only those days on which progeny were produced, the mean was 12.98 ± 0.79 (N = 39) per day. (TIF) [file pone.0300426.s002.tif]

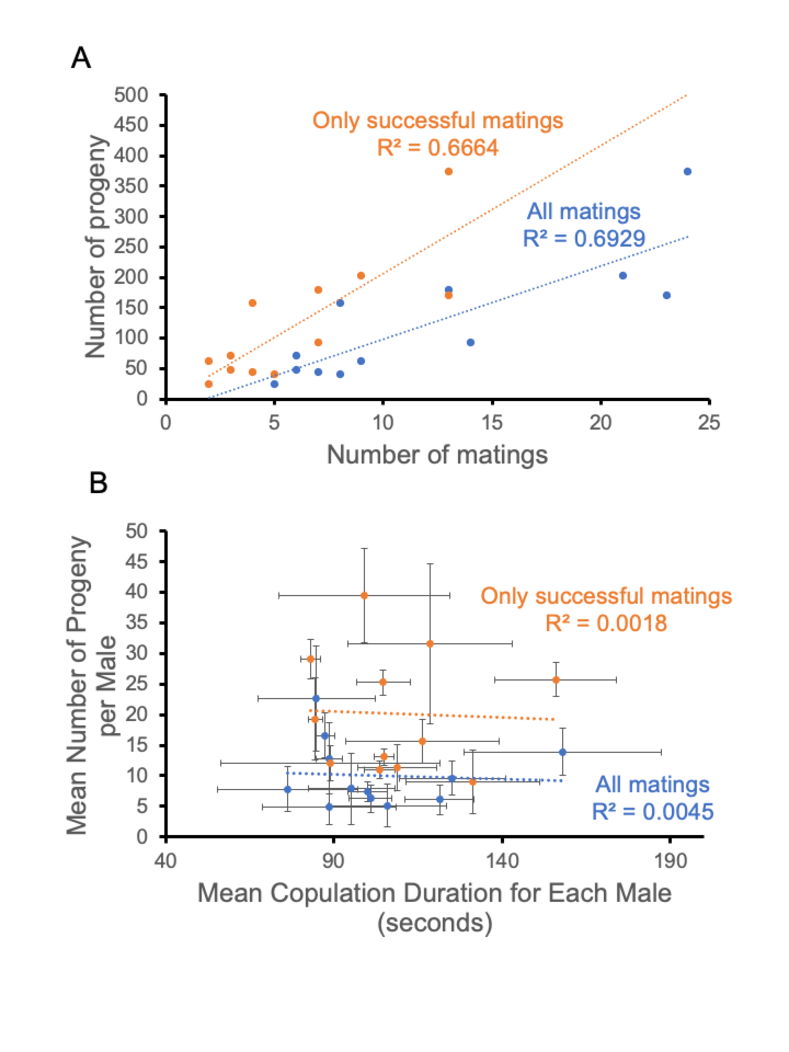

Supplement: S3 Fig — A. The total number of matings for each male (N = 12) over four days was positively associated with the total number of progeny produced by each male when all matings were included (blue, P = 0.0008) or when only those producing progeny were included (orange, P = 0.001). B. Progeny production was not related to copulation duration for individual males. Data shown are the mean for each male ± standard error. (TIF) [file pone.0300426.s003.tif]
